# Supplementary material for: Yra2 regulates proteolysis of Cse4 to prevent its mislocalization to non-centromeric regions for chromosomal stability in budding yeast
Source: bioRxiv. 2026 Aug 12:2026.08.11.744167. Preprint. [Version 1] doi: 10.64898/2026.08.11.744167 (PMC13640705; doi:10.64898/2026.08.11.744167)

bioRxiv preprint doi: https://doi.org/10.64898/2026.08.11.744167; this version posted August 12, 2026. The copyright holder for this preprint (which was not certified by peer review) is the author/funder. This article is a US Government work. It is not subject to copyright under 17 USC 105 and is also made available for use under a CC0 license.

Supplemental Figures

Fig. S1. The N-terminus of Cse4 contributes to the *GALCSE4* induced SDL in *yra2Δ* strains. Wild-type with vector (YMB12793) or *GALcse4Δ129* (YMB12924) and the isogenic *yra2Δ* with vector (YMB12791) or *GALcse4Δ129* (YMB12927) were grown to logarithmic phase, five-fold serial dilutions were prepared and plated on SC-Ura plates containing either glucose (2%) or galactose+raffinose (2% each) at 25°C.

Fig. S2. The steady state levels of histone H4 and Cse4 are not affected across different strains examined in this study (Related to Fig. 5).

Fig. S3. Endogenous Cse4 is enriched in chromatin in *yra2Δ* strains. a) Levels of chromatin bound endogenously expressed Cse4 in *yra2Δ* strain. Whole cell extracts (WCE), soluble (SOL) and chromatin fraction (CF) from wild-type (YMB10574), and *yra2Δ* (YMB13044) expressing HA-Cse4 from its native promoter at the endogenous locus were analyzed by western blotting using anti-HA (HA-Cse4), anti-Tub2, and anti-H2B antibodies. Tub2 and histone H2B were used as markers for soluble and chromatin fractions, respectively. b) Chromatin enrichment of Cse4 is significantly increased in *yra2Δ* strain. Enrichment as a ratio of Cse4 in chromatin fraction over input and normalized to H2B were determined from intensity values calculated from western blots using Image J (SCHNEIDER *et al.* 2012). Average±SE from three biological replicates is shown. **p* value <0.05, Student's t-test.

bioRxiv preprint doi: https://doi.org/10.64898/2026.08.11.744167; this version posted August 12, 2026. The copyright holder for this preprint (which was not certified by peer review) is the author/funder. This article is a US Government work. It is not subject to copyright under 17 USC 105 and is also made available for use under a CC0 license.

Fig. S1

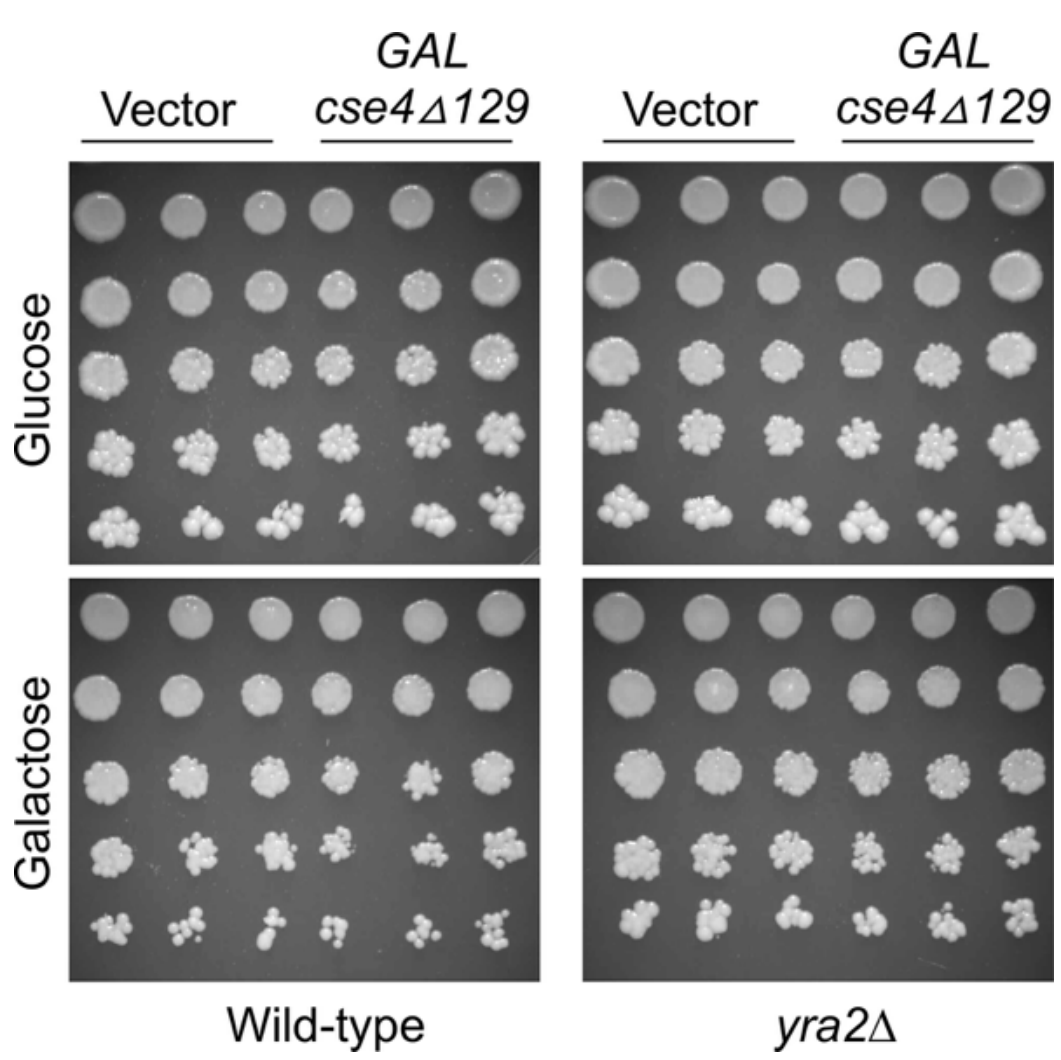

bioRxiv preprint doi: https://doi.org/10.64898/2026.08.11.744167; this version posted August 12, 2026. The copyright holder for this preprint (which was not certified by peer review) is the author/funder. This article is a US Government work. It is not subject to copyright under 17 USC 105 and is also made available for use under a CC0 license.

Fig. S2

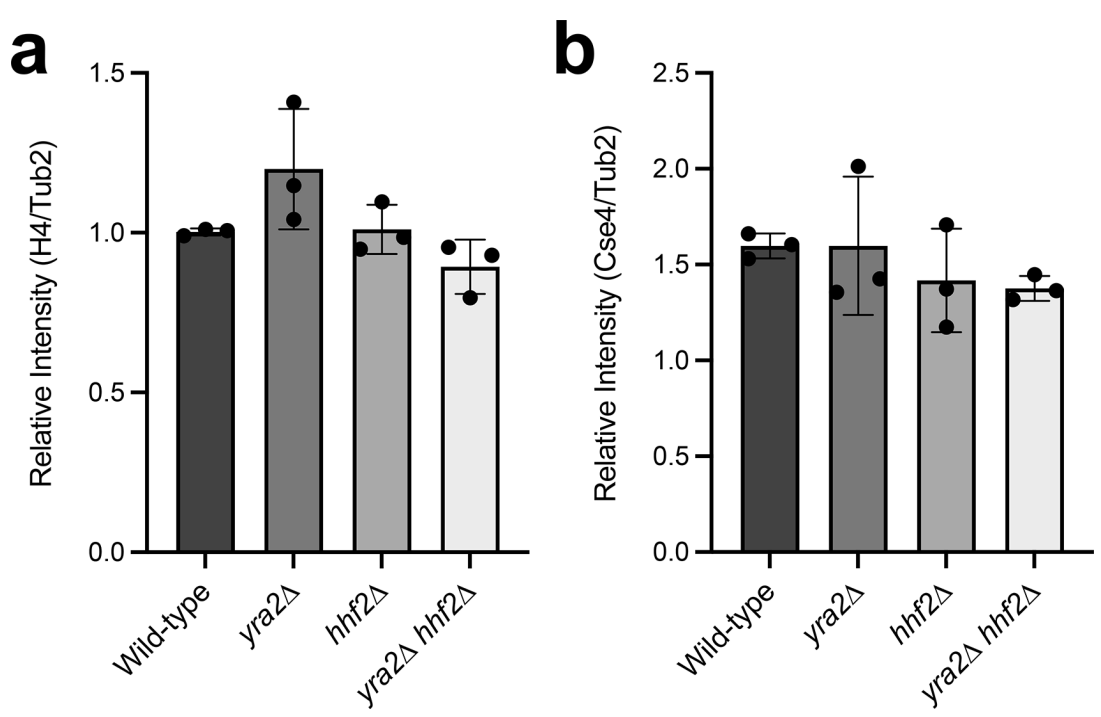

Fig. S3

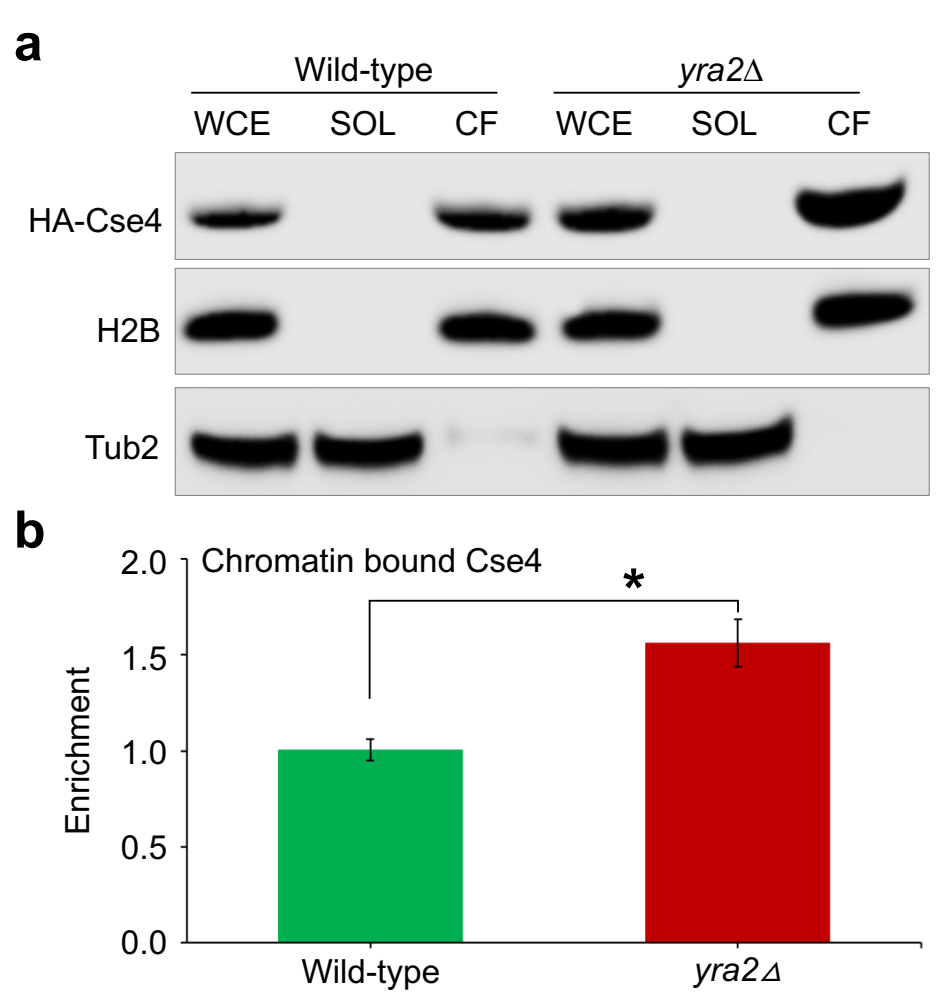

Supplement: Supplementary file 1 [file NIHPP2026.08.11.744167v1-supplement-1.pdf]
